# Ditopic Receptors Based on Dihomooxacalix[4]arenes Bearing Phenylurea Moieties With Electron-Withdrawing Groups for Anions and Organic Ion Pairs

**DOI:** 10.3389/fchem.2019.00758

**Published:** 2019-11-08

**Authors:** Alexandre S. Miranda, Defne Serbetci, Paula M. Marcos, José R. Ascenso, Mário N. Berberan-Santos, Neal Hickey, Silvano Geremia

**Affiliations:** ^1^Centro de Química Estrutural, Faculdade de Ciências da Universidade de Lisboa, Lisbon, Portugal; ^2^Centro de Química-Física Molecular, Institute of Nanoscience and Nanotechnology (IN) and IBB-Institute for Bioengineering and Biosciences, Instituto Superior Técnico, Universidade de Lisboa, Lisbon, Portugal; ^3^Faculdade de Farmácia da Universidade de Lisboa, Lisbon, Portugal; ^4^Centro de Química Estrutural, Instituto Superior Técnico, Lisbon, Portugal; ^5^Department of Chemical and Pharmaceutical Sciences, Centre of Excellence in Biocrystallography, University of Trieste, Trieste, Italy

**Keywords:** dihomooxacalix[4]arenes, phenylurea anion receptors, biogenic amine hydrochlorides, ditopic receptors, proton NMR titrations, UV-Vis absorption studies, fluorescence studies, X-ray diffraction

## Abstract

Two bidentate dihomooxacalix[4]arene receptors bearing phenylurea moieties substituted with electron-withdrawing groups at the lower rim via a butyl spacer (CF_3_-Phurea **5b** and NO_2_ Phurea **5c**) were obtained in the cone conformation in solution, as shown by NMR. The X-ray crystal structure of **5b** is reported. The binding affinity of these receptors toward several relevant anions was investigated by ^1^H NMR, UV-Vis absorption in different solvents, and fluorescence titrations. Compounds **5b** and **5c** were also tested as ditopic receptors for organic ion pairs, namely monoamine neurotransmitters and trace amine hydrochlorides by ^1^H NMR studies. The data showed that both receptors follow the same trend and, in comparison with the unsubstituted phenylurea **5a**, they exhibit a significant enhancement on their host-guest properties, owing to the increased acidity of their urea NH protons. NO_2_-Phurea **5c** is the best anion receptor, displaying the strongest complexation for F^−^, closely followed by the oxoanions BzO^−^, AcO^−^, and HS${\text{O}}_{4}^{-}$. Concerning ion pair recognition, both ditopic receptors presented an outstanding efficiency for the amine hydrochlorides, mainly **5c**, with association constants higher than 10^9^ M^−2^ in the case of phenylethylamine and tyramine.

## Introduction

Anion recognition by synthetic receptors continues to attract much attention, as documented by the reviews published recently (Evans and Beer, 2014; Busschaert et al., 2015; Gale et al., 2016). Anions play essential roles in numerous biological systems, as well as in many environmental and industrial processes.

Macrocyclic compounds have been developed as anion receptors, in which the interactions are mainly established by hydrogen bond donor groups, such as ureas and thioureas, incorporated in the macrocycle scaffolds. These receptors are able to recognize anions with different geometries through effective and directional H-bonds. However, to bind a charged species these monotopic receptors need to overcome the tendency of the target ion to form an ion pair with its counter ion, especially in non-polar solvents. Thus, heteroditopic receptors, molecules capable of simultaneously bind both ions of a given ion pair, have been obtained and are an emerging area in supramolecular chemistry (Kim and Sessler, 2010; McConnell and Beer, 2012). These systems have important applications, as membrane transport agents, and in salt extraction and solubilisation. The binding ability of these ditopic receptors toward organic ion pairs, namely ammonium and amino acid salts, has been more investigated in the last years. Alkylammonium moieties are a constant presence in compounds of biological interest, such as biogenic amines, trace amines and amino acids (Mutihac et al., 2011).

Calixarenes are a particularly attractive class of macrocyclic compounds owing to the relatively ease functionalization of both the upper and the lower rims, and to the presence of a pre-organized cavity available in different sizes and conformations (Gutsche, 2008). These compounds have been largely used as anion receptors. In particular, calix[4]arenes (Quinlan et al., 2007; Babu et al., 2009; Curinova et al., 2009; De Solis et al., 2015; Klejch et al., 2016; Rezankova et al., 2017) and calix[6]arenes (Hamon et al., 2008; Nehra et al., 2016) bearing phenylurea moieties incorporating electron-withdrawing groups, such as NO_2_ and CF_3_, have been investigated. These groups are expected to increase the acidity of the urea NH protons, thus enhancing the anion binding ability of the receptors. Calixarenes have also been used as building blocks for the construction of ditopic receptors able of simultaneous binding of anions and cations. Examples of such receptors based on calix[4] (Pescatori et al., 2009), calix[5] (Capici et al., 2010), and mainly calix[6]arenes (Hamon et al., 2008; Lascaux et al., 2010; Cornut et al., 2015; Moerkerke et al., 2017) are reported in the literature.

In the course of our studies on anion binding by disubstituted dihomooxacalix[4]arenes (calix[4]arene analogs in which one CH_2_ bridge is replaced by one CH_2_OCH_2_ group) (Marcos, 2016) with phenylurea units (Marcos et al., 2014a,b), we were interested in determine the enhancement on the anion binding affinity of the receptors by the introduction of electron-withdrawing groups. Along with this research, the phenylurea derivatives were also evaluated as ditopic receptors (Gattuso et al., 2015). They combine in the same molecule two different binding sites, i.e., an ureido anionic site and a hydrophobic cavity suitable for organic cations.

In this paper we describe the synthesis of two new disubstituted dihomooxacalix[4]arenes bearing, via a butyl spacer, *para* CF_3_- (**5b**) or NO_2_-phenylurea (**5c**) moieties, at the 1,3-positions of the lower rim. The host-guest properties of these receptors, obtained in the cone conformation, toward several relevant anions were established by proton NMR and UV-Vis absorption titrations in chloroform (or dichloromethane) and acetonitrile. Some photophysical properties of these receptors (due to their intrinsic fluorescence) were evaluated and, in a few cases, fluorescence studies were also performed to investigate the calixarene binding affinity. These dihomooxa derivatives were also tested as heteroditopic receptors for *n*-alkylammonium chlorides, and monoamine neurotransmitters and trace amine hydrochlorides by proton NMR studies. The results are compared to those obtained with the analog unsubstituted phenylurea (**5a**). The solid state structure of **5b** is also presented.

## Results and Discussion

### Synthesis and Structural Analysis

**Scheme 1:**
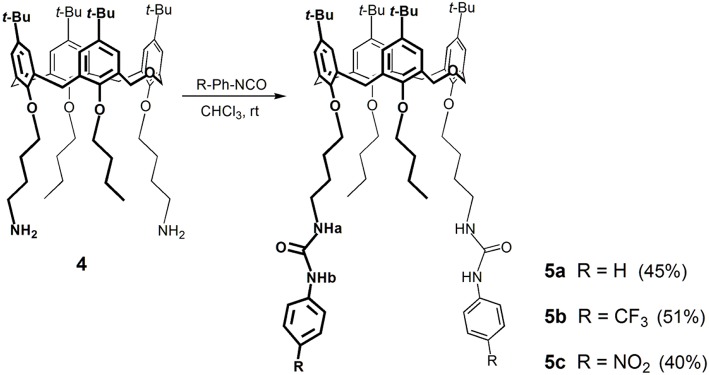
Synthesis of the phenylurea receptors **5a**–**5c**.

Previously, we have reported the synthesis of a lower rim 1,3-disubstituted dihomooxacalix[4]arene receptor containing two phenylurea moieties and two *n*-butyl groups (Marcos et al., 2014a). Following this line of research, we synthesized two new ureido-dihomooxacalix[4]arenes bearing CF_3_ or NO_2_ groups at the *p*-position of the phenylurea moiety, via a butyl spacer. The binding ability of these receptors is expected to be increased by the introduction of the electron-withdrawing groups. Thus, the alkylation reaction of the parent *p-tert*-butyldihomooxacalix[4]arene (**1**) with 4-bromobutyronitrile and K_2_CO_3_ gave the dicyano-dihydroxy compound **2**, which was further alkylated with *n*-butyl iodide and NaH, yielding the dicyano-dibutoxy derivative **3**. Reduction of the cyano groups with NaBH_4_/CoCl_2_ afforded diamine **4**. These reactions and products were already described (Marcos et al., 2014a). Diamine **4** reacted with *p*-(trifluoromethyl)- or *p*-nitro-phenylisocyanate to yield the corresponding *p*-CF_3_-phenylurea **5b** and *p*-NO_2_-phenylurea **5c**, in the cone conformation (Scheme 1).

**Figure 1:**
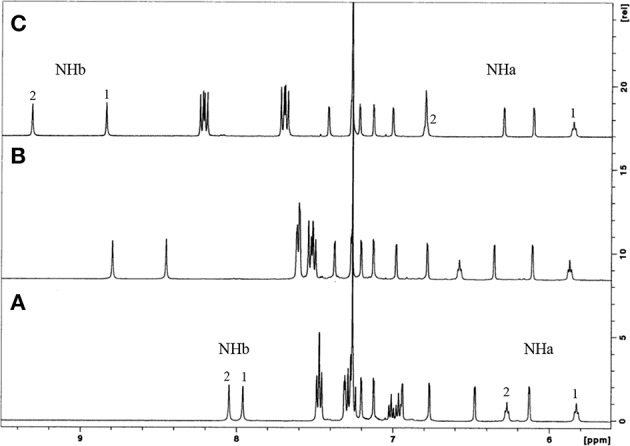
^1^H NMR partial spectra (500 MHz, CDCl_3_, 25°C) of: **(A)** Phurea **5a**, **(B)** CF_3_-Phurea **5b**, and **(C)** NO_2_-Phurea **5c**.

These receptors are inherently chiral, as indicated by their NMR spectra in CDCl_3_. The proton spectra show four singlets for the *tert*-butyl groups, five AB quartets for the CH_2_ bridge protons, four pairs of doublets for the aromatic protons, and two triplets and two singlets for the NHa and NHb protons, respectively. The aromatic and NH region of the three phenyl ureas is shown in Figure 1. Increasing downfield shifts for the NH protons, mainly the NHb, can be observed from Phurea **5a** to NO_2_-Phurea **5c**, indicating the increased acidity of these protons. Moreover, the proton spectra display two triplets and several multiplets for the methyl and methylene protons of the butyl spacers and *n*-butyl groups. The ^13^C spectra exhibit three ArCH_2_Ar resonances in the range 28.8–31.5 ppm, indicative of a cone conformation (Jaime et al., 1991). The proton assignments were confirmed by COSY spectra.

**Figure 2:**
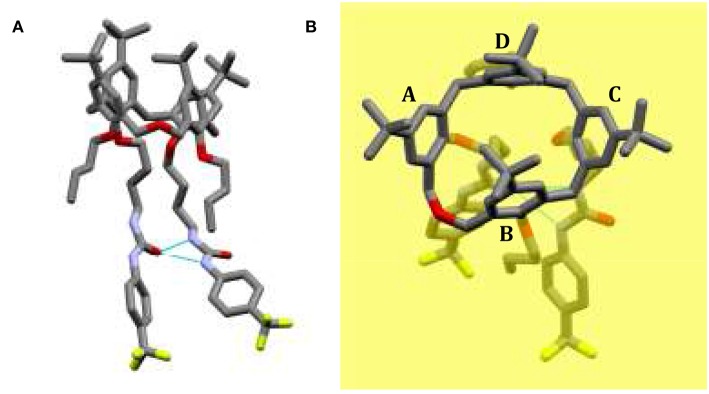
Solid state structure of CF_3_-Phurea **5b**. **(A)** The molecule shows a cone conformation, with the phenylureido moieties involved in an intramolecular N–H···O bifurcated hydrogen bond. **(B)** Orthogonal view of **5b** with respect to the dihomooxacalixarene mean plane (yellow) defined by the methylene bridging groups.

Small colorless single crystal needles were obtained by slow evaporation of a chloroform solution containing compound **5b**. The X-ray structure was determined using synchrotron radiation with crystals frozen at 100 K. The asymmetric unit of the monoclinic crystal (space group P2_1_/c) is composed of one molecule of **5b** and a disordered co-crystallized chloroform solvent molecule with a total occupancy factor of 0.7. The dihomooxacalixarene macrocycle adopts the expected cone conformation, producing an inherently chiral molecule due to the 1,3-substitution pattern on the lower rim (Figure 2, rings A and C). The centrosymmetric space group implies the presence of a racemic mixture of the two enantiomers in the crystals. The mean planes of the two ureido substituted phenyl rings (A and C) make large outward (dihedral) angles of 125° and 131°, respectively, with respect to the dihomooxacalixarene mean plane, defined by the methylene bridging groups (Figure 2B). With regard to the butoxy substituted phenyl rings, the one connected with the homooxo bridge (B) shows a mean plane of the phenyl ring inclined inwards with a dihedral angle of 66°. As a result, its *p-tert*-butyl group partially occupies the calixarene cavity. The facing butoxy substituted phenyl ring (D) is inclined slightly outwards, with a dihedral angle of 101°. Consistently with previous reports, the ureido groups form an intramolecular N–H···O bifurcated hydrogen bond (Marcos et al., 2014b; Augusto et al., 2018). In this case the H-bond is rather asymmetric, with the N···O distance of the NH directly bonded to the phenyl ring, *para* to the electron-withdrawing CF_3_ group, shorter with respect to the other N···O distance (2.89 vs. 3.06 Å). The relative orientations of the skeletons of the NCON ureido moieties show a mean plane dihedral angle of 28°, while the terminal phenyl groups form dihedral angles of about 20° (Ring A) and 40° (Ring C) with respect to their corresponding planar ureido groups. The overall result is that these phenyl groups show a dihedral angle of about 74° between their mean planes (Figure 2).

**Figure 3:**
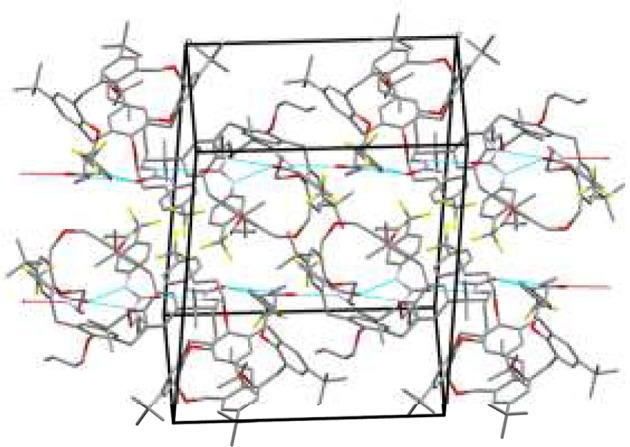
Crystal packing of **5b** shows two chains of bifurcated N–H···O hydrogen bonds from NH donors to O acceptor. The one-dimensional chains propagate along the c axis in antiparallel fashion.

The crystal packing shows that the ureido groups are involved in an intermolecular N–H···O hydrogen bonds network. More specifically, one-dimensional chains of bifurcated H-bonds, formed by iso-orientated calixarenes (generated by crystallographic glide planes), are propagated antiparallel along the c-axis (Figure 3). The resulting intermolecular N···O distances show that these bifurcated intermolecular H-bonds are even more asymmetric than the intramolecular H-bonds. In this case the NH group directly bound to the phenyl ring forms a weaker H-bond with the carbonyl oxygen of a symmetry related molecule in comparison to the second NH group (N···O distances are 3.14 and 2.85 Å, respectively). The antiparallel chains are related by crystallographic screw axes and stacked along the b-axis with the calix cups facing the CF_3_-phenyl rings (Figure 3). The chloroform molecules at partial occupancy fill the voids created by the crystal packing of the calixarene molecules.

The comparison with the solid state structure of **5a** containing two crystallographic independent molecules, previously reported (Gattuso et al., 2015), shows an analogous crystal packing arrangement, despite the significant differences in the unit cells and the different space groups. However, in **5a** the orientations of the NHCONH ureido moieties are quite different in the two crystallographic independent molecules, being almost parallel in one (similar to the conformation found in **5b**) and orthogonally oriented in the other (Gattuso et al., 2015). For each independent molecules of **5a**, the cone macrocycle adopts a similar conformation to **5b**, in which the *p-tert*-butyl groups of the B rings lean into the cavity. More specifically, for the molecule with the almost parallel orientation of the ureido groups, the four dihedral angles formed by the mean planes of the phenyl rings and the dihomooxacalixarene bridging mean plane are within two degrees of the molecule found in **5b**. On the other hand, the **5a** molecule having an orthogonal orientation of the NHCONH ureido moiety shows a more open cone conformation (121, 74, 137, 97° for A, B, C, and D angles, respectively). This comparison suggests that the openness of the cone is to some degree related to the conformation and reciprocal orientation of the NHCONH ureido moieties.

### Photophysical Properties

Owing to the intrinsic fluorescence of receptors **5**, and to evaluate the changes caused by the introduction of the substituent groups at the *p*-position of the phenylurea ring, some photophysical properties of **5a** and **5b** were determined, following previous studies (Miranda et al., 2017).

**Figure 4:**
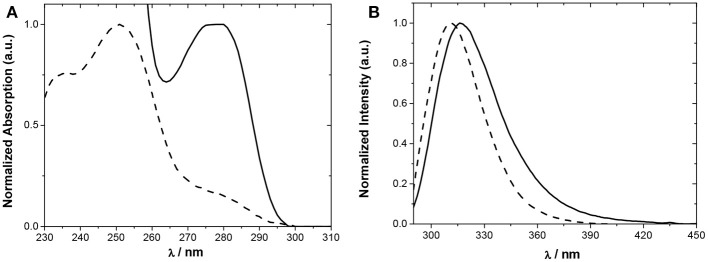
Normalized spectra of **5a** (5.0 × 10^−5^ M; solid line) and **5b** (5.0 × 10^−6^ M; dashed line) in CH_2_Cl_2_. **(A)** Absorption and **(B)** emission.

The absorption and steady-state fluorescence spectra of Phurea **5a** and CF_3_-Phurea **5b** in dichloromethane are shown in Figure 4. The compounds present a well-defined absorption in the UV region, exhibiting a blue shift of 28 nm for **5b** (Figure 4A). The same trend is observed in the emission spectrum, the normalized spectra being again similar for both compounds (Figure 4B).

Relevant photophysical properties of the two Phureas are collected in Table 1. Stokes shifts were calculated as the difference between the excitation and the emission peak wavelengths. The results show that CF_3_-Phurea **5b** presents a higher value compared to **5a**. The fluorescence lifetimes (τ_f_) and quantum yields (ϕ_F_) were also determined. Overall, fluorescence lifetimes and yields do not change much upon *para* substitution, the fluorescence quantum yields are significant (0.2–0.6) and lifetimes moderate (1–2 ns). The quantum yield decreases and the lifetime increases upon *para* substitution, both effects resulting mainly from a decrease of the radiative rate constant (*k*_r_) (Table 1).

**Table 1:** Photophysical properties of Phureas **5a** and **5b** in CH_2_Cl_2_ at 25°C.

	**λ_**max, abs**_ (nm)**	**λ_***max, em***_ (nm)**	**ε (M^**−1**^ cm^**−1**^)**	**Stokes shift^a^ (nm)**	**τ_**f**_ (ns)**	***ϕ_F_***	***k*_**r**_ (ns^**−1**^)**	***k*_**nr**_ (ns^**−1**^)**
**5a**	278	316	5.0 × 10^3^	38	1.15	0.59^b^	0.51	0.36
**5b**	250	310	7.1 × 10^4^	60	1.57	0.21^c^	0.13	0.50

a *Compute as λ_max,em_− λ_max,abs_*.

b *Against quinine sulfate ϕ_F_ = 0.60 in HCl 0.1 M*.

c *Against tryptophan ϕ_F_ = 0.12 in water*.

### Anion Recognition

#### Proton NMR Studies

The binding properties of bidentate CF_3_-Phurea **5b** and NO_2_-Phurea **5c** toward several relevant anions of different geometries (spherical, linear, trigonal planar, and tetrahedral) were studied in CDCl_3_ through ^1^H NMR titrations using tetrabutylammonium (TBA) salts. The association constants (as log *K*_ass_) reported in Table 2 were determined using the WinEQNMR2 program (Hynes, 1993) and following the urea NH chemical shifts. In a few cases where those protons became broad or disappeared, the association constants were calculated through the complexation induced shifts of the aromatic protons of the calixarene skeleton.

**Table 2:** Association constants (log *K*_ass_)^a^ of dihomooxa ureas **5a**-**5c** determined by ^1^H NMR in CDCl_3_ at 25°C.

	**Spherical**				**Linear**		**Trigonal planar**			**Tetrahedral**		
	**F^**−**^**	**Cl^**−**^**	**Br^**−**^**	**I^**−**^**	**CN^**−**^**	**SCN^**−**^**	**N${\text{O}}_{3}^{-}$**	**AcO^**−**^**	**BzO^**−**^**	**HS${\text{O}}_{4}^{-}$**	**H_**2**_P${\text{O}}_{4}^{-}$**	**Cl**${\text{O}}_{4}^{-}$
I. Radius/Å^b^	1.33	1.81	1.96	2.20	1.91	2.13	1.79	2.32	—	1.90	2.00	2.40
Phurea **5a**^c^	3.10	2.73	2.23	1.59	2.71	1.90	2.42	2.88	2.93	2.58	2.69	1.65
CF_3_-Phurea **5b**	3.48	3.12	2.68	2.18	3.13	2.18	2.68	3.34	3.46	3.07	3.15	2.04
NO_2_-Phurea **5c**	3.88	3.65	3.07	2.65	3.66	2.67	3.15	3.67	3.83	3.61	3.49	2.37

a *Estimated error <10%*.

b *Data quoted in Marcus (1997)*.

c *Data taken from Marcos et al. (2014a)*.

**Figure 5:**
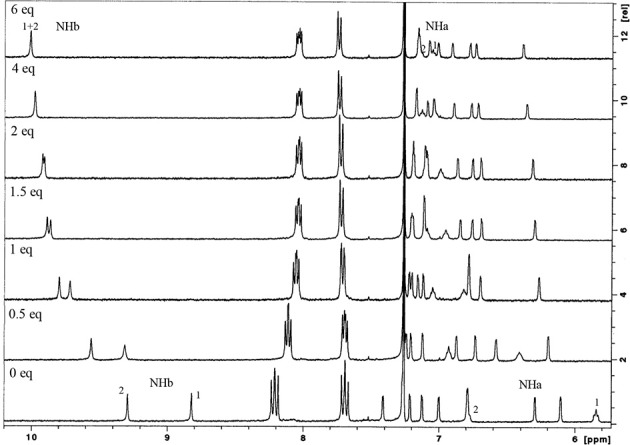
Partial ^1^H NMR spectra of NO_2_-Phurea **5c** (500 MHz, CDCl_3_, 25°C) with several equiv of TBA Cl.

Hydrogen bonding interactions between the anions and the urea groups of the receptors were clearly evidenced by the downfield shifts of their NH protons, as shown in Figure 5. In all the studied cases, the complexation process occurs under fast exchange conditions on the NMR time scale at room temperature. The titration curves obtained (Figure S1) indicate the formation of 1:1 host-guest complexes. This stoichiometry was also confirmed by Job plots (Figure S2).

The anion binding results obtained by proton NMR titrations (Table 2) show that both phenylureas bearing the electron-withdrawing groups CF_3_ and NO_2_ are better receptors than Phurea **5a** (with no substituents), due to the increased acidity of their NH protons. All the receptors follow the same trend: the association constants decrease in general with decreasing anion basicity. The data show that NO_2_-Phurea **5c** is the best anion receptor, exhibiting high association constants. Among the spherical halides, **5c** displays the strongest complexation for F^−^ (log *K*_ass_ = 3.88). The results with F^−^ showed no evidence for the formation of the H${\text{F}}_{2}^{-}$ species (Amendola et al., 2006, 2010; Babu et al., 2009) Although the acidity of Phureas **5b** and **5c** is higher compared to that of **5a**, the solvent used, chloroform, is a weakly competitive one, contributing to stabilize the H-bond complexes. Moreover, small downfield and upfield shifts for the *ortho* and *meta* protons, respectively, of the phenylurea groups of **5c** were also observed (Figure S3), corroborating the expected effects for the formation of hydrogen-bonding complexes (Amendola et al., 2010). In the case of the pseudo-halides, the more basic CN^−^ anion was complexed with higher selectivity with respect to SCN^−^ (*K*_ass_ CN^−^/*K*_ass_ SCN^−^ = 8.9 and 9.8 for **5b** and **5c**, respectively). With regard to the planar oxoanions, receptors **5b** and especially **5c** show a very efficient binding toward the carboxylates BzO^−^ and AcO^−^ (log *K*_ass_ = 3.83 and 3.67, respectively, for **5c**). As observed before with **5a** and with other dihomooxa ureas (Marcos et al., 2014b; Teixeira et al., 2017), there is a slight inversion of the basicity order. π staking interactions may contribute to this slight increase of the BzO^−^ complexation over that of AcO^−^. The inorganic oxoanions, HS${\text{O}}_{4}^{-}$ and H_2_P${\text{O}}_{4}^{-}$, are also tightly bound by these receptors.

#### UV-Vis Absorption and Fluorescence Studies

The binding properties of CF_3_- and NO_2_-Phureas (**5b** and **5c**) were complemented through UV-Vis absorption titrations. Thus, the interactions between these receptors and some selected anions of different geometries (F^−^, Cl^−^, Br^−^, N${\text{O}}_{3}^{-}$, AcO^−^, BzO^−^, HS${\text{O}}_{4}^{-}$, and H_2_P${\text{O}}_{4}^{-}$) as TBA salts were studied in chloroform (or dichloromethane) and acetonitrile solvents.

**Figure 6:**
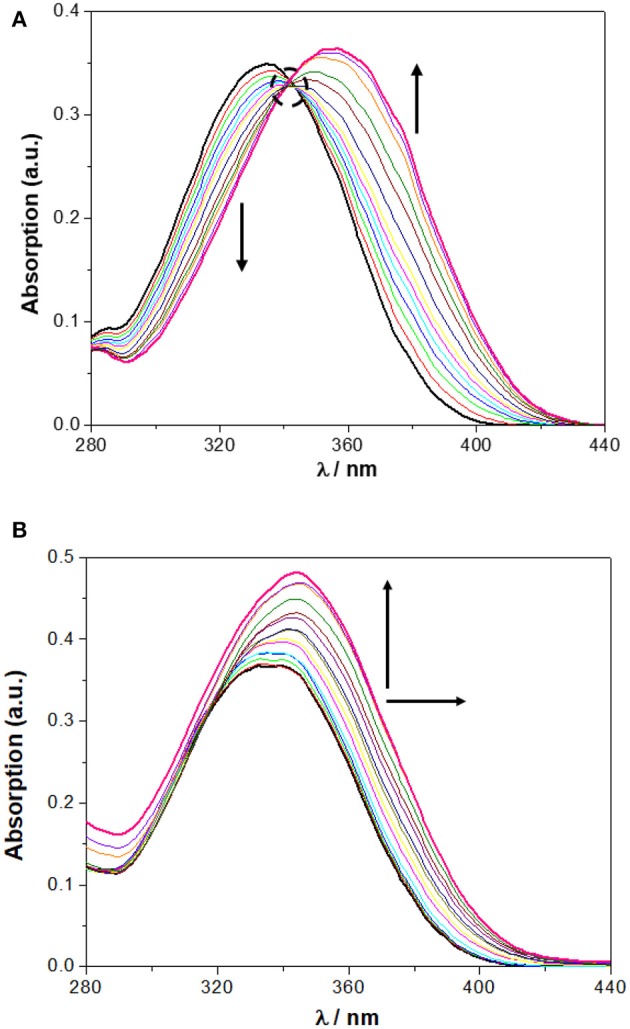
Changes in the UV spectra of NO_2_-Phurea **5c** (1.0 × 10^−5^ M) upon addition of TBA F (up to 10 equiv) in: **(A)** CHCl_3_ and **(B)** MeCN. The dotted circle indicates the isosbestic point. The arrows indicate the decreasing or increasing amounts of salt and maximum displacement.

**Figure 7:**
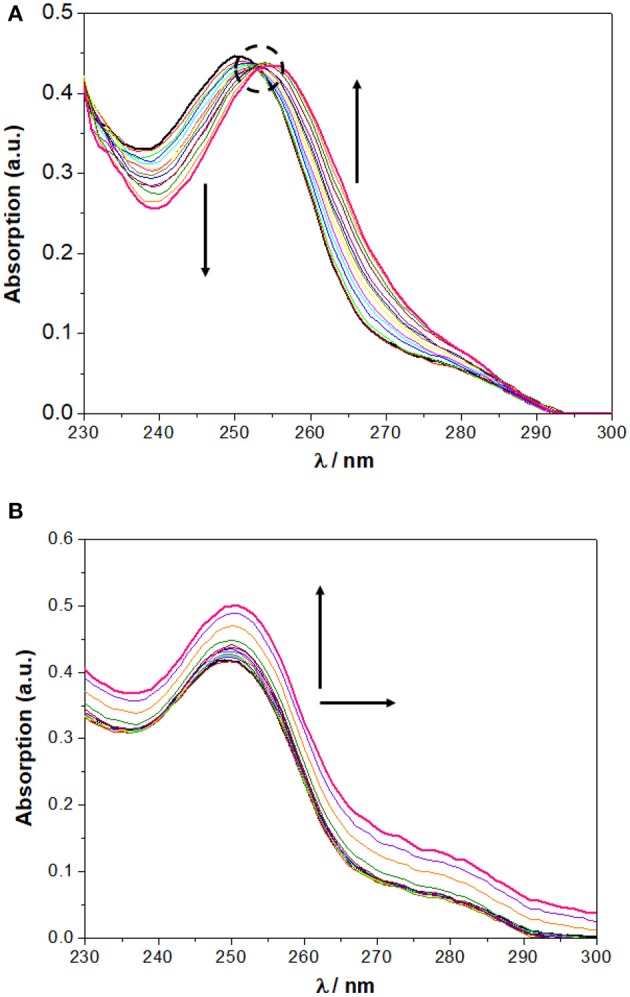
Changes in the UV spectra of CF_3_-Phurea **5b** (5.0 × 10 ^−6^ M) upon addition of TBA BzO (up to 10 equiv) in: **(A)** CH_2_Cl_2_ and **(B)** MeCN. The dotted circle indicates the isosbestic point. The arrows indicate the decreasing or increasing amounts of salt and maximum displacement.

In chloroform, NO_2_-Phurea **5c** displays an absorption band centered at approximately 335 nm. Upon addition of increasing amounts of F^−^ ion, this band decreases in intensity while a new one is progressively formed, reaching a maximum at 356 nm (red shift of 21 nm) and exhibiting an isosbestic point at 341 nm, which reveals the existence of only two species (Figure 6A). Similar absorption spectral changes were observed for the carboxylates AcO^−^ and BzO^−^, both leading to red shifts of 15 nm and presenting isosbestic points, as well as for the inorganic oxoanions HS${\text{O}}_{4}^{-}$ and H_2_P${\text{O}}_{4}^{-}$, although to a smaller extent. In the case of addition of the spherical Cl^−^ and Br^−^ anions, and the planar N${\text{O}}_{3}^{-}$, successive increases of the absorption were recorded, but with almost no shifts in their maxima. Similar absorption spectra were obtain for NO_2_-Phureido-calix[4]arene analogs (Babu et al., 2009; Curinova et al., 2009). CF_3_-Phurea **5b** exhibits a similar behavior in dichloromethane. In this case it was not possible to use chloroform solvent due to absorption overlapping with urea **5b**. The addition of increasing amounts of F^−^, AcO^−^, BzO^−^, and H_2_P${\text{O}}_{4}^{-}$ anions to a solution of **5b** leads to a decrease of the intensity of its absorption peak at 250 nm, while a new band appears and progressively moves to longer wavelength. Isosbestic points can also be observed, as illustrated in Figure 7A for the BzO^−^ anion. The other anions studied showed no new band formation at higher wavelengths; only a gradual increase of the absorption band centered at 250 nm was observed as the anion concentration increased. In acetonitrile, both receptors showed identical behaviors toward all the anions: a successive increase of the absorption in the presence of the anions, with no significant modification of their band shapes (Figures 6B, 7B).

In all cases, the spectral variations were sufficiently important to allow the determination of the corresponding binding constants. The data presented in Table 3 show a stronger complexation in chloroform (or dichloromethane in the case of **5b**) than in acetonitrile for both ureas, in agreement with the competitiveness of the solvents. The association constants in both solvents are higher than those obtained by NMR, but follow the same trend: F^−^, AcO^−^, and BzO^−^ are the best bound anions. The UV concentrations are more than 200 times less than those used in the NMR titrations, and this fact influences the association constants. The more diluted solutions used in the UV experiments favor the dissociation of the salts, thereby providing a higher concentration of the anions available for binding and resulting in higher association constants.

**Table 3:** Association constants (log *K*_ass_)^a^ of dihomooxa ureas **5b** and **5c** determined by UV-Vis absorption at 25°C.

	**Spherical**				**Trigonal planar**			**Tetrahedral**	
	**Solvent**	**F^**−**^**	**Cl^**−**^**	**Br^**−**^**	**N${\text{O}}_{3}^{-}$**	**AcO^**−**^**	**BzO^**−**^**	**HS${\text{O}}_{4}^{-}$**	**H_**2**_P${\text{O}}_{4}^{-}$**
CF_3_-Phurea **5b**	CH_2_Cl_2_	4.57	4.02	3.66	3.72	4.54	4.65	3.93	3.93
	MeCN	4.10	3.81	3.54	3.50	4.03	4.20	3.81	3.60
NO_2_-Phurea **5c**	CHCl_3_	4.74	4.30	3.90	3.97	4.65	4.72	4.19	4.24
	MeCN	4.52	4.04	3.61	3.63	4.13	4.30	3.77	3.83

*Estimated error <10%*.

The N${\text{O}}_{3}^{-}$ and HS${\text{O}}_{4}^{-}$ anion binding constants with CF_3_-Phurea **5b** were also determined by fluorescence in CH_2_Cl_2_. The data obtained (log *K*_ass_: N${\text{O}}_{3}^{-}$ = 3.87 and HS${\text{O}}_{4}^{-}$ = 4.00) are higher, as expected, than those obtained for **5a** (log *K*_ass_ = 3.5 and 3.7) (Miranda et al., 2017), but follow the same trend. The results are also similar to those obtained by UV-Vis absorption (Table 3), indicating that fluorescence can also be a good method for the determination of the receptor-anion association constants.

#### Organic Ion Pair Recognition

Based on our earlier good results obtained with Phurea **5a** (Gattuso et al., 2015), dihomooxa receptors **5b** and **5c** have also been tested as heteroditopic receptors for *n*-propyl and *n*-butylammonium chlorides in a preliminary study to evaluate their complexation behavior. Besides the presence of a hydrophobic cavity and an anionic binding site in close proximity, CF_3_- and NO_2_-Phureas displayed an enhancement of their binding efficiency for Cl^−^ anion compared to that of **5a** (almost one log unity in the case of **5c**), being expected a higher positive effect on the salt complexation.

**Figure 8:**
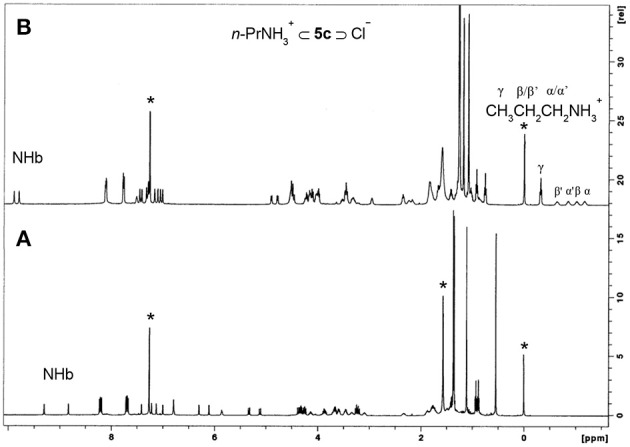
^1^H NMR spectra (500 MHz, CDCl_3_, 25°C) of: **(A)** NO_2_-Phurea **5c**, **(B) 5c** + 1 eq of *n*-PrNH_2_·HCl. ^*^Denotes residual solvent signals.

Proton NMR titrations were performed in CDCl_3_ by adding increasing amounts (up to two equiv) of the salts to solutions of the receptors **5b** and **5c**. Three sets of resonances corresponding to the free and complexed receptors, and to the guest bound to the host were observed on addition of the first aliquot of the salts. The alkylammonium cation inclusion inside the dihomooxa cavity is demonstrated by the appearance of the alkyl group resonances in the negative region of the spectrum. On the other side, simultaneous chloride binding to the urea moiety is shown by the downfield shifts observed for all the NH protons, indicating complexation of the anion through hydrogen-bond interactions (Figure 8). This Figure also shows the pairs of enantiotopic hydrogen atoms of the α- and β-CH_2_ groups of the included guest displaying non-equivalent signals owing to the chiral environment of the host. The free host signals disappeared with subsequent additions of the salts. This binding process occurs under slow exchange condition on the NMR time scale. All these host-guest pairs studied displayed percentages of complexation higher than 95%, corresponding to association constants higher than 10^9^ M^−2^.

**Figure 9:**
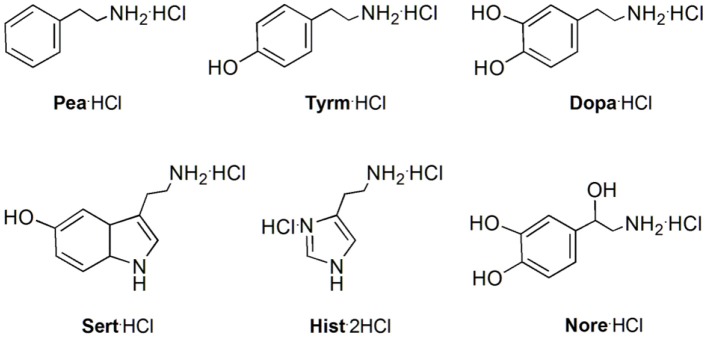
Structures of the monoamine neurotransmitters and trace amine hydrochlorides studied: 2-phenylethylamine (Pea·HCl), tyramine (Tyrm·HCl), dopamine (Dopa·HCl), serotonin (Sert·HCl), histamine (Hist·2HCl), and norepinephrine (Nore·HCl).

**Figure 10:**
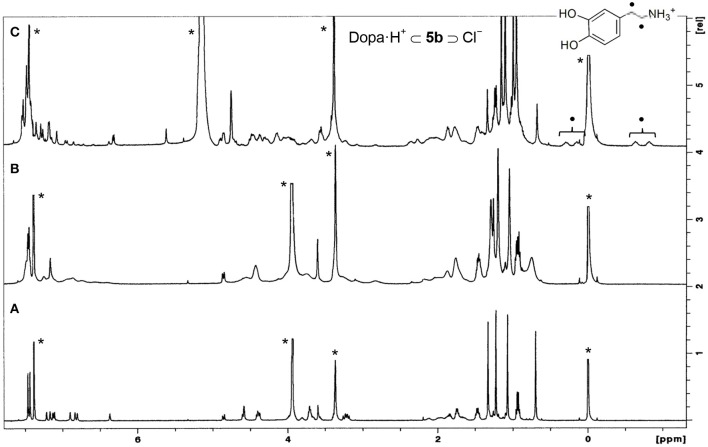
^1^H NMR spectra (500 MHz, CDCl_3_/CD_3_OD, 5:1, v/v) of: **(A)** [CF_3_-Phurea **5b**] = 1.0 mM at 298 K, **(B)** [**5b**] = [Dopa·HCl] = 1.0 mM at 298 K, and **(C)** [**5b**] = [Dopa·HCl] = 1.0 mM at 233 K. ^*^Residual solvent signals.

These ureas were then tested in the recognition of the monoamine neurotransmitter and trace amine hydrochlorides shown in Figure 9. The studies were done in a CDCl_3_/CD_3_OD solvent mixture (5:1, v/v) for a better solubility of the guests. The addition of one equiv. of the guests to a solution of the hosts at room temperature (Figure 10A) induced a large broadening of all signals, indicating a strong host-guest interaction (Figure 10B). To obtain a clear interpretation of the spectra, it was however necessary to lower the temperature to 233 K. As illustrated in Figure 10C for **5b** with dopamine·HCl, four high field signals for the α- and β-CH_2_ protons of the guest were observed, showing their inclusion inside the asymmetric cavity of the host. The slow exchange rate between the free and the complexed receptors allowed the determination of the percentages of complexation and of the corresponding association constants, by direct integration of the peaks. The data (Table 4) show that both ureas display an outstanding efficiency toward the biogenic amines, being as expected better than Phurea **5a**. Both ureas present very high percentages of complexation and association constants, even preventing us from calculating *K*_ass_ for phenylethylamine and also tyramine in the case of NO_2_-Phurea **5c**. This urea is more efficient than CF_3_-Phurea **5b**, but the latter is slightly more selective. Ureas **5b** and **5c** display a similar affinity trend, comparable with **5a**, showing some selectivity for Pea and Tyrm and no interaction with histamine and norepinephrine. As mentioned before (Gattuso et al., 2015), it seems that the less bulky Pea and Tyrm guests fit better inside the dihomooxa cavity.

**Table 4:** Percentages of complex formation and corresponding association constants, *K*_ass_ (M^−2^)^a^.

	**Pea·HCl**	**Tyrm·HCl**	**Dopa·HCl**	**Sert·HCl**	**Hist·2HCl**	**Nore·HCl**
Phurea **5a**^b^	86% 41,000	85% 36,000	67% 6,300	61% 4,100	c	c
CF_3_-Phurea **5b**	>95% >10^9^	91% 1,23,000	79% 18,000	81% 22,000	c	c
NO_2_-Phurea **5c**	>95% >10^9^	>95% >10^9^	87% 51,000	86% 42,000	c	c

a *CDCl_3_/CD_3_OD (5:1, v/v), 233 K; Estimated error ≤15%*.

b *Data taken from Gattuso et al. (2015)*.

*^c^ No complexation observed*.

## Conclusions

Two new 1,3-disubstituted dihomooxacalix[4]arene receptors containing *para* CF_3_- (**5b**) or NO_2_-phenylurea (**5c**) moieties on the lower rim linked by a butyl spacer were obtained in the cone conformation in solution. The X-ray structure of **5b** was obtained and confirms the cone conformation with one *tert*-butyl-phenyl group oriented toward the center of the cup. The 1,3-substitution pattern on the lower rim results in inherently chiral molecules, present as racemic mixture in the centrosymmetric crystals. In the crystal structure, the ureido groups are involved in asymmetric intra- and inter-molecular bifurcated H-bonds.

The anion binding affinity of **5b** and **5c** was established by ^1^H NMR, UV-Vis and fluorescence studies. These receptors form 1:1 complexes with anions of different geometries through hydrogen bonding. Comparing to the unsubstituted phenylurea **5a**, CF_3_-Phurea and especially NO_2_-Phurea showed, as expected, a relevant enhancement on their binding efficiency, due to the increased acidity of their urea NH protons. Compound **5c** displayed the strongest complexation for F^−^ (log *K*_ass_ = 3.88), closely followed by the oxoanions BzO^−^, AcO^−^, and HS${\text{O}}_{4}^{-}$. The association constants obtained by UV-Vis absorption titrations followed the same trend of those obtained by NMR, and were higher in CHCl_3_ (or CH_2_Cl_2_) than in MeCN, according to the competitiveness of the solvents.

As heteroditopic receptors, both compounds exhibited remarkable ion pair recognition, displaying very high association constants for the monoamine neurotransmitters tyramine, dopamine and serotonin, and the trace amine phenylethylamine hydrochlorides. The more efficient ditopic receptor **5c** presented *K*_ass_ higher than 10^9^ M^−2^ in the case of Pea and Tyrm, which turns it into a potential candidate for biogenic amine chemosensors in biological fluids.

## Experiment

### Synthesis

All chemicals were reagent grade and were used without further purification. Chromatographic separations were performed on Merck silica gel 60 (particle size 40–63 μm, 230–400 mesh). Melting points were measured on a Stuart Scientific apparatus and are uncorrected. FTIR spectra were recorded on a Shimadzu Model IRaffinity-1 spectrophotometer. ^1^H and ^13^C NMR spectra were recorded on a Bruker Avance III 500 MHz spectrometer, with TMS as internal reference. The conventional COSY experiment was collected as 256×2 K complex points. Elemental analysis was determined on a Fisons EA 1108 microanalyser.

#### Procedure for the Synthesis of Ureas 5b and 5c

To a solution of **4** (0.71 g, 0.762 mmol) in CHCl_3_ (35 mL) was added 1.53 mmol of the appropriate isocyanate. The mixture was stirred at room temperature under N_2_ for 4 h. Evaporation of the solvent yielded the crude products which were purified as described below.

#### 7,13,19,25-Tetra-Tert-Butyl-27,29-Bis[[N′-(p-Trifluoromethylphenylureido)butyl]oxy]-28,30-dibutoxy-2,3-dihomo-3-oxacalix[4]arene (5b)

Flash chromatography (SiO_2_, eluent CH_2_Cl_2_/MeOH, 99.7:0.3): it was obtained in 51% yield (0.51 g); mp 151–152°C; IR (KBr) 3,350 cm^−1^ (NH), 1,647 cm^−1^ (CO); ^1^H NMR (CDCl_3_, 500 MHz) δ 0.55, 1.10, 1.34, 1.37 [4s, 36H, C(CH_3_)], 0.89, 0.93 (2t, 6H, *J* = 7.4 Hz, CH_3_), 1.33–1.54 (m, 8H, OCH_2_CH_2_C*H*_2_CH_3_ and OCH_2_CH_2_C*H*_2_CH_2_NH_a_), 1.77, 1.89, 2.31 (3m, 8H, OCH_2_C*H*_2_CH_2_CH_3_ and OCH_2_C*H*_2_CH_2_CH_2_NH_a_), 3.16, 3.37, 3.59 (3m, 4H, OCH_2_CH_2_CH_2_C*H*_2_NH_a_), 3.20, 4.28 (ABq, 2H, *J* = 12.6 Hz, ArCH_2_Ar), 3.22, 4.26 (ABq, 2H, *J* = 14.1 Hz, ArCH_2_Ar), 3.24, 4.38 (ABq, 2H, *J* = 12.5 Hz, ArCH_2_Ar), 3.45, 3.71, 3.90, 4.07 (4m, 4H, OC*H*_2_CH_2_CH_2_CH_2_NH_a_), 3.59, 3.66, 3.82 (3m, 4H, OC*H*_2_CH_2_CH_2_CH_3_**)**, 4.32, 5.26 (ABq, 2H, *J* = 12.4 Hz, CH_2_OCH_2_), 4.33, 5.04 (ABq, 2H, *J* = 12.7 Hz, CH_2_OCH_2_), 5.87, 6.57 (2t, 2H, NH_a_), 6.11, 6.35, 6.78, 6.98, 7.12, 7.20, 7.27, 7.37 (8d, 8H, ArH), 7.50, 7.53 (2d, 4H, Ph-H_*o*_), 7.60, 7.61 (2d, 4H, Ph-H_*m*_), 8.45, 8.79 (2s, 2H, NH_b_); ^13^C NMR (CDCl_3_, 125.8 MHz) δ 13.9, 14.1 [O(CH_2_)_3_*C*H_3_], 19.3, 19.4 (OCH_2_CH_2_*C*H_2_CH_3_), 25.0, 25.5, 25.8, 28.8 (OCH_2_*C*H_2_*C*H_2_CH_2_NH_a_), 28.9. 31.0, 31.3 (ArCH_2_Ar), 31.2, 31.4, 31.6, 31.7 [C(*C*H_3_)], 32.2, 32.6 (OCH_2_*C*H_2_CH_2_CH_3_), 33.6, 33.9, 34.21, 34.22 [*C*(CH_3_)], 39.3, 39.4 (OCH_2_CH_2_CH_2_*C*H_2_NH_a_), 71.7, 71.9 (CH_2_OCH_2_), 73.2, 74.8, 75.0, 75.6 (O*C*H_2_CH_2_CH_2_CH_2_NH_a_ and O*C*H_2_CH_2_CH_2_CH_3_), 117.7, 1,178, 123.7, 125.4, 125.6, 126.0, 126.1, 126.3, 126.49, 126.52, 126.9, 129.5 (ArH), 123.3, 124.1 (q, *J* = 32 Hz, CF_3_), 128.6, 130.2, 131.8, 132.7, 133.1, 134.3, 134.6, 136.2, 143.1, 143.4, 144.4, 145.0, 145.1, 145.7, 152.7, 153.1, 153.9, 155.3 (Ar), 155.6, 156.7 (CO). Anal. Calcd for C_77_H_100_N_4_O_7_F_6_: C, 70.73; H, 7.71; N, 4.28. Found: C, 70.78; H, 8.01; N, 4.02.

#### 7,13,19,25-Tetra-Tert-Butyl-27,29-Bis[[N′-(p-Nitrophenylureido)Butyl]oxy]-28,30-dibutoxy-2,3-dihomo-3-oxacalix[4]arene (5c)

Flash chromatography (SiO_2_, eluent CH_2_Cl_2_/MeOH, 99.7:0.3) followed by recrystallization from CH_2_Cl_2_/*n*-hexane: it was obtained in 40% yield (0.38 g); mp 157–159°C; IR (KBr) 3,367 cm^−1^ (NH), 1,647 cm^−1^ (CO); ^1^H NMR (CDCl_3_, 500 MHz) δ 0.54, 1.11, 1.35, 1.37 [4s, 36H, C(CH_3_)], 0.88, 0.93 (2t, 6H, *J* = 7.4 Hz, CH_3_), 1.38–1.50 (m, 4H, OCH_2_CH_2_C*H*_2_CH_3_**)**, 1.48, 1.76, 1.87, 2.34 (4m, 12H, OCH_2_C*H*_2_C*H*_2_CH_2_NH_a_ and OCH_2_C*H*_2_CH_2_CH_3_**)**, 3.09, 3.34, 3.45, 3.59 (4m, 4H, OCH_2_CH_2_CH_2_C*H*_2_NH_a_), 3.20, 4.26 (ABq, 2H, *J* = 12.7 Hz, ArCH_2_Ar), 3.23, 4.24 (ABq, 2H, *J* = 14.0 Hz, ArCH_2_Ar), 3.26, 4.37 (ABq, 2H, *J* = 12.6 Hz, ArCH_2_Ar), 3.45, 3.59, 3.66, 4.14 (4m, 4H, OC*H*_2_CH_2_CH_2_CH_2_NH_a_), 3.66, 3.87 (2m, 4H, OC*H*_2_CH_2_CH_2_CH_3_**)**, 4.31, 5.33 (ABq, 2H, *J* = 12.3 Hz, CH_2_OCH_2_), 4.33, 5.12 (ABq, 2H, *J* = 12.3 Hz, CH_2_OCH_2_), 5.85, 6.79 (2t, 2H, NH_a_), 6.10, 6.29, 6.79, 7.00, 7.12, 7.21, 7.26, 7.41 (8d, 8H, ArH), 7.68, 7.71 (2d, 4H, Ph-H_*o*_), 8.20, 8.23 (2d, 4H, Ph-H_*m*_), 8.83, 9.31 (2s, 2H, NH_b_); ^13^C NMR (CDCl_3_, 125.8 MHz) δ 13.9, 14.1 [O(CH_2_)_3_*C*H_3_], 19.3, 19.4 (OCH_2_CH_2_*C*H_2_CH_3_), 24.8, 25.2, 25.7, 28.81 (OCH_2_*C*H_2_*C*H_2_CH_2_NH_a_), 28.84, 31.1, 31.4 (ArCH_2_Ar), 31.2, 31.4, 31.5, 31.7 [C(*C*H_3_)], 32.2, 32.6 (OCH_2_*C*H_2_CH_2_CH_3_), 33.6, 34.0, 34.24, 34.23 [*C*(CH_3_)], 39.3 (2C) (OCH_2_CH_2_CH_2_*C*H_2_NH_a_), 71.7, 72.1 (CH_2_OCH_2_), 73.9, 74.9, 75.1, 75.6 (O*C*H_2_CH_2_CH_2_CH_2_NH_a_ and O*C*H_2_CH_2_CH_2_CH_3_), 117.0, 117.1, 123.6, 125.3, 125.56, 125.60, 125.63, 126.0, 126.1, 126.6, 126.9, 130.0 (ArH), 128.3, 129.7, 131.6, 132.7, 133.1, 134.3, 134.6, 136.2, 141.6, 142.1, 144.4, 145.1, 145.2, 145.8, 146.1, 146.7, 152.7, 153.2, 153.8, 154.9 (Ar), 155.7, 156.3 (CO). Anal. Calcd for C_75_H_100_N_6_O_11_: C, 71.40; H, 7.99; N, 6.66. Found: C, 70.99; H, 7.99; N, 6.48.

### Determination of the Crystallographic Structure of Compound 5b

Small colorless single crystal needles were obtained by slow evaporation of a chloroform solution containing compound **5b**. The single crystals investigated were very small (0.01, 0.01, 0.05 mm) and synchrotron radiation was necessary to obtain a dataset suitable to solve the structure. The data was also collected with frozen crystal at 100 K. Despite these provisions, the best small crystal showed poor diffraction data with a maximum resolution of 0.98 Å. Data collection was carried out at the Macromolecular crystallography XRD1 beamline of the Elettra synchrotron (Trieste, Italy), employing the rotating-crystal method with a Dectris Pilatus 2M area detector. Single crystals were dipped in PEG200 cryoprotectant, mounted on a loop and flash-frozen under a liquid nitrogen stream at a 100 K. Diffraction data were indexed and integrated using the XDS package (Kabsch, 2010a), while scaling was carried out with XSCALE (Kabsch, 2010b). The structure was solved using the SHELXT program (Sheldrick, 2015) and structure refinement was performed with SHELXL-14 (Sheldrick, 2008), operating through the WinGX GUI (Farrugia, 2012) by full-matrix least-squares (FMLS) methods on F^2^.

The structure was solved using the SHELXT program (Sheldrick, 2015). The asymmetric unit of the monoclinic crystal (space group P2_1_/c) is composed of one molecule of **5b** and disordered co-crystallized chloroform solvent molecule with a total occupancy factor of 0.7. The chloroform molecule shows disorder over two positions, which were isotropically refined at 0.4/0.3 partial occupancies. All other non-hydrogen atoms were anisotropically refined at full occupancy. Hydrogen atoms were added at the calculated positions and refined using the riding model. Crystallographic data and refinement details are reported in Table S1.

### ^1^H NMR Titrations

The anion association constants (as log *K*_ass_) were determined in CDCl_3_ by ^1^H NMR titration experiments. Several aliquots (up to 10 equiv) of the anion solutions (as tetrabutylammonium salts) were added to 0.5 mL solution of the receptors (2.5 × 10^−3^ – 5 × 10^−3^ M) directly in the NMR tube. The spectra were recorded after each addition of the salts, and the temperature of the NMR probe was kept constant at 25°C. For each anion-receptor system titrations were repeated at least two times. The association constants were evaluated using the WinEQNMR2 program (Hynes, 1993) by following the urea NH chemical shifts. When possible, *K*_ass_ was calculated as a mean value of the four NH chemical shifts. The Job plots were performed keeping the total concentration in the same range as before. Concerning ion-pair recognition experiments, the percentage of complex formation, necessary for the calculation of the corresponding *K*_ass_, was determined by direct ^1^H NMR integration of the free and complexed resonances of the hosts and/or the guests, present at equilibrium. The samples were prepared by mixing aliquots of stock solutions of the host (600 μl) and guests (60 μl) to obtain a final equimolar host-guest solution of 1.0 × 10^−3^ M. Details related to these experiments have already been described (Gattuso et al., 2015).

### UV-Vis Absorption and Fluorescence Studies

Absorption and fluorescence studies were done using an UV-3101PC UV-Vis-NIR spectrophotometer and a Fluorolog F112A fluorimeter in right-angle configuration, respectively. The association constants were determined in CHCl_3_ (or CH_2_Cl_2_) and MeCN by UV-Vis absorption spectrophotometry at 25°C. A few anion complexation studies were also done by steady-state fluorescence in CH_2_Cl_2_. The spectra were recorded between 230 and 300 nm or 280–440 nm, in the case of NO_2_-Phurea **5c**, and using quartz cells with an optical path length of 1 cm. Several aliquots (up to 10 equiv) of the anion solutions (as TBA salts) were added to a 2 mL solution of the receptors (5.0 × 10^−6^ – 5.0 × 10^−5^ M) directly in the cell. The spectral changes were interpreted using the HypSpec 2014 program (Gans et al., 1996). Details concerning the photophysical properties determination are given in the Supporting Information.

## Data Availability Statement

The datasets generated for this study are available on request to the corresponding author.

## Author Contributions

AM synthesized and characterized the compounds, carried out the UV-Vis absorption, and fluorescence studies. DS contributed to the synthesis, characterization, and UV-Vis titrations of one of the compounds. PM designed the study, performed the NMR experiments, their analysis and interpretation, and wrote the manuscript. JA carried out some NMR experiments, their analysis and interpretation, contributed also to the writing, and critical review of the manuscript. MB-S performed the analysis, interpretation, and writing of the photophysics results. NH did the crystallization and structure determination by X-ray diffraction, the analysis, interpretation, and manuscript preparation for structural data. SG performed the analysis, interpretation, and manuscript preparation for structural data.

### Conflict of Interest

The authors declare that the research was conducted in the absence of any commercial or financial relationships that could be construed as a potential conflict of interest.
