# Supplementary material for: Ditopic Receptors Based on Dihomooxacalix[4]arenes Bearing Phenylurea Moieties With Electron-Withdrawing Groups for Anions and Organic Ion Pairs
Source: Front Chem. 2019 Nov 8;7:758. doi: 10.3389/fchem.2019.00758 (PMC6857623; doi:10.3389/fchem.2019.00758)
Supplement: Supplementary file 1 [file Data_Sheet_1.docx]

**Supporting Information**

**Ditopic Receptors Based on Dihomooxacalix[4]arenes Bearing Phenylurea Moieties with Electron-Withdrawing Groups for Anions and Organic Ion Pairs**

Alexandre S. Miranda,^ab^ Defne Serbetci,^a^ Paula M. Marcos,*^ac^

José R. Ascenso,^d^ Mário N. Berberan-Santos,^b^ Neal Hickey,^e^ Silvano Geremia^e^

^a^Centro de Química Estrutural, Faculdade de Ciências da Universidade de Lisboa, Edifício C8, 1749-016 Lisboa, Portugal. E-mail: pmmarcos@fc.ul.pt

^b^Centro de Química-Física Molecular, Institute of Nanoscience and Nanotechnology (IN) and IBB-Institute for Bioengineering and Biosciences, Instituto Superior Técnico, Universidade de Lisboa,

1049-001 Lisboa, Portugal

^c^Faculdade de Farmácia da Universidade de Lisboa, Av. Prof. Gama Pinto, 1649-003 Lisboa, Portugal

^d^Instituto Superior Técnico, CQE, Complexo I, Av. Rovisco Pais, 1049-001 Lisboa, Portugal

^e^Centre of Excellence in Biocrystallography, Department of Chemical and Pharmaceutical Sciences, University of Trieste, via L. Giorgieri 1, 34127 Trieste, Italy

**Table of contents**

1. Titration curves of **5b** with TBA salts in CDCl_3_

2. Job’s plot based on ^1^H NMR data for **5b** + Br^−^ and **5c** + H_2_PO_4_^−^

3. Aromatic region of ^1^H NMR spectra of NO_2_-Phurea **5c** with several equiv of TBA F

4. Crystallographic data and refinement details

5. Photophysical properties determination

6. ^1^H NMR spectra of **5b** and **5c**

7. ^13^C NMR spectra of **5b** and **5b**

**Figure** **S1.** Titration curves of CF_3_-Phurea **5b** with TBA salts in CDCl_3_.

a)

[Complex] (mM)

Molar fraction of CF_3_-Phurea **5b**

b)

[Complex] (mM)

Molar fraction of NO_2_-Phurea **5c**

**Figure** **S2.** Job plot based on ^1^H NMR data for (a) CF_3_-Phurea **5b** + Br^−^, (b) NO_2_-Phurea **5c** + H_2_PO_4_^−^; total concentration 2.5 × 10^–3^ M in CDCl_3_.


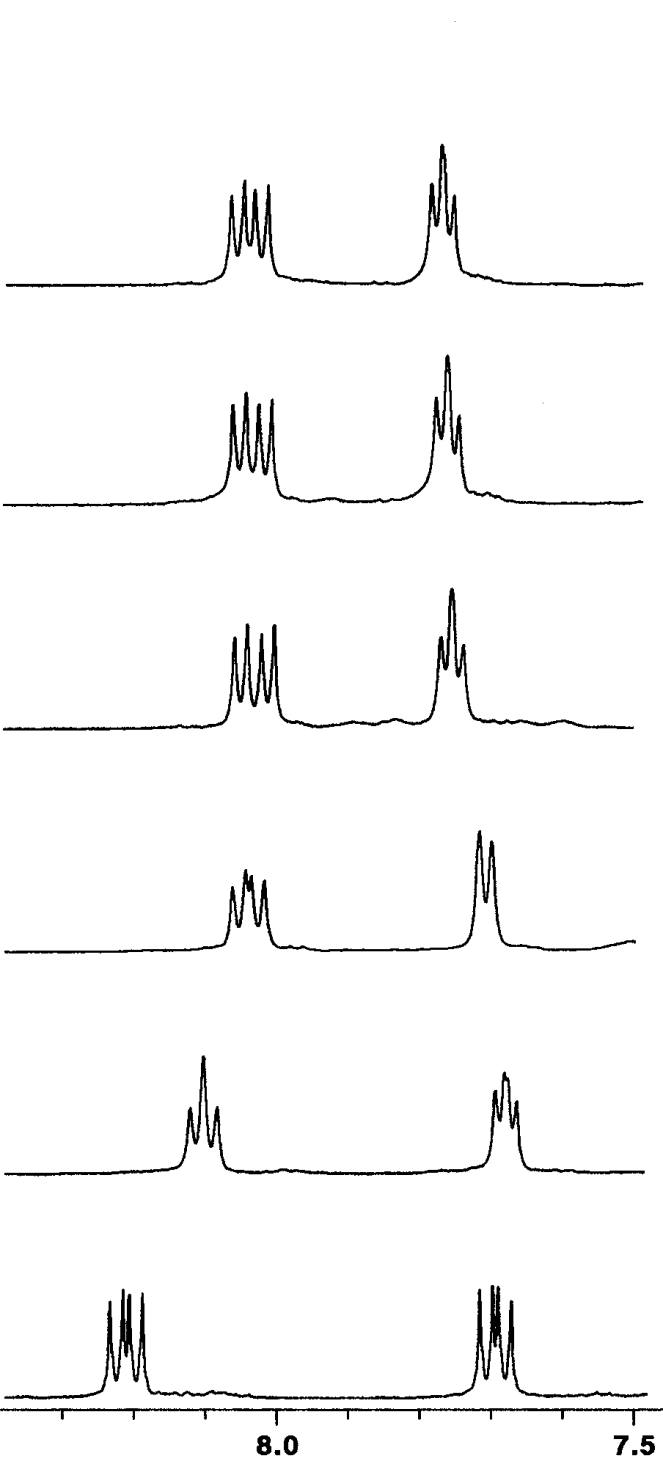


2 eq

0 eq

0.5 eq

1 eq

3 eq

5 eq

H*_orto_*

H*_meta_*





**Figure S3.** Partial aromatic region (H*_orto_* and H*_meta_*) of ^1^H NMR spectra of NO_2_-Phurea **5c** (500 MHz, CDCl3, 25 °C) with several equiv of TBA F.

Table S1. Crystal data and structure refinement for compound 5b

|  | **5b** |
| --- | --- |
| Empirical formula | (C_77_H_100_N_4_O_7_F_6_), 0.7(CHCl_3_) |
| Formula weight | 1391.37 |
| Temperature (K) | 100(2) |
| Wavelength (Å) | 0.7 |
| Crystal system | Monoclinic |
| Space group | *P* 2_1_/c |
| Unit cell dimensions (Å, °) | *a* = 23.60(3), *α* = 90 |
|  | *b* = 18.26(2), *β* = 107.75(6) |
|  | *c* = 18.47(3), *γ* = 90 |
| Volume (Å^3^) | 7581(19) |
| Z | 4 |
| *ρ* _calcd_ (g/cm^3^) | 1.219 |
| *μ* (mm^–1^) | 0.150 |
| F(000) | 2962 |
| Reflections collected | 28013 |
| Independent reflections | 8476 [R(int) = 0.0775] |
| Data / restraints / parameters | 8476 / 0 / 893 |
| GooF | 1.023 |
| Final *R* indices [I>2σ(I)] | *R*_1_ = 0.0615, *wR*_2_ = 0.1538 |
| R indices (all data) | *R*_1_ = 0.1077, *wR*_2_ = 0.1809 |
| CCDC code | 1941590 |

**Photophysical properties determination**

Molar absorption coefficients of calixarenes in dichloromethane were determined from absorbance vs concentration plots. Time-resolved fluorescence intensity decays were obtained using a single-photon timing method with laser excitation and microchannel plate detection, with the set-up already described (Menezes et al., 2013). The excitation wavelength used was at the maximum absorption of the calixarenes and the emission wavelengths at the maximum emission, using a front-face geometry. The timescale changed between 8.1 ps per channel for Phurea **5a** and 9.8 ps for CF_3_-Phurea. Decay data analysis with a sum of exponentials was achieved by means of a Microsoft Excel spreadsheet specially designed for lifetime analysis that considers the convolution with the IRF (Berberan-Santos, 2009). Fluorescent quantum yields were measured using quinine sulfate as reference (*Ф*_F_ = 0.60 in HCl 0.1 M) for compounds **5a** and tryptophan (*Ф*_F_ = 0.12 in water) for Phurea **5b** (Brouwer, 2011). To prevent inner filter effects during quantum yield measurements, the absorbance of the samples and the references was kept below 0.1 at the excitation wavelength. Fluorescence quantum yields were computed using equation 1 (Valeur and Berberan-Santos, 2012):

$$\Phi=\Phi_{ref}\left( \frac{I}{I_{ref}} \right)\left( \frac{A_{ref}}{A} \right)\left( \frac{n^{2}}{n_{ref}^{2}} \right) 1$$

Where $\Phi$ is quantum yield, *I* is the integrated fluorescence emission intensity, *A* is the absorbance at the excitation wavelength, and *n* is the refractive index of the solvent (CH_2_Cl_2_ and water).

**References**

Berberan-Santos, M.N. (2009). unpublished.

Brouwer, A.M. (2011). Standards for photoluminescence quantum yield measurements in solution (IUPAC Technical Report). *Pure Appl. Chem.* 83, *12*, 2213-2228. doi: 10.1351/PAC-REP-10-09-31

Menezes, F., Fedorov, A., Baleizão, C., Valeur, B., and Berberan-Santos, M.N. (2013). Methods for the analysis of complex fluorescence decays: sum of Becquerel functions versus sum of exponentials. *Methods Appl. Fluoresc.* 1, 015002. doi: 10.1088/2050-6120/1/1/015002

Valeur, B., Berberan-Santos, M.N. (2012). Molecular Fluorescence, Wiley-VCH, 2^nd^ edn.


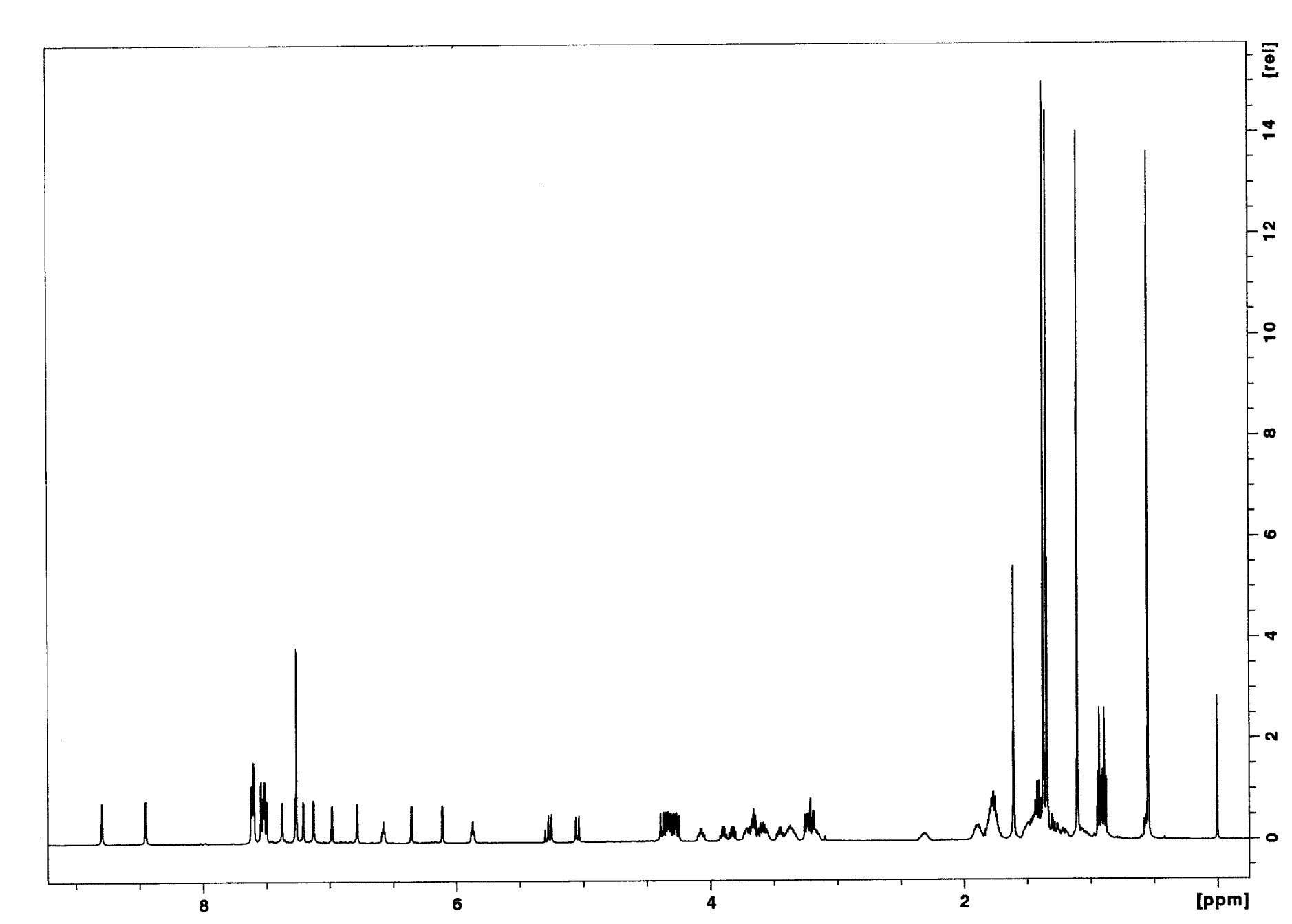


**Figure S4.** ^1^H NMR spectrum of CF_3_-Phurea **5b**.


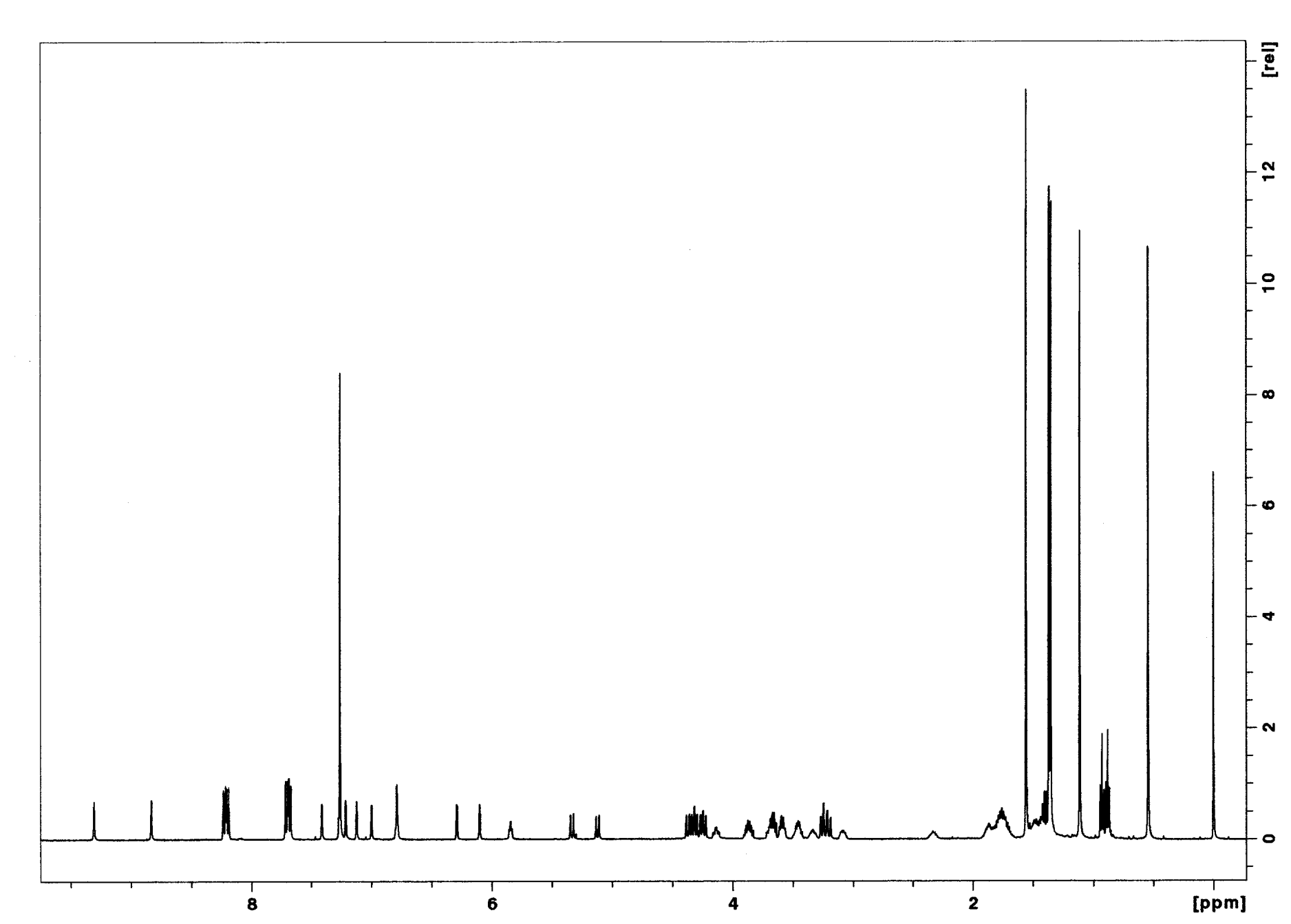


**Figure S5.** ^1^H NMR spectrum of NO_2_-Phurea **5c**.


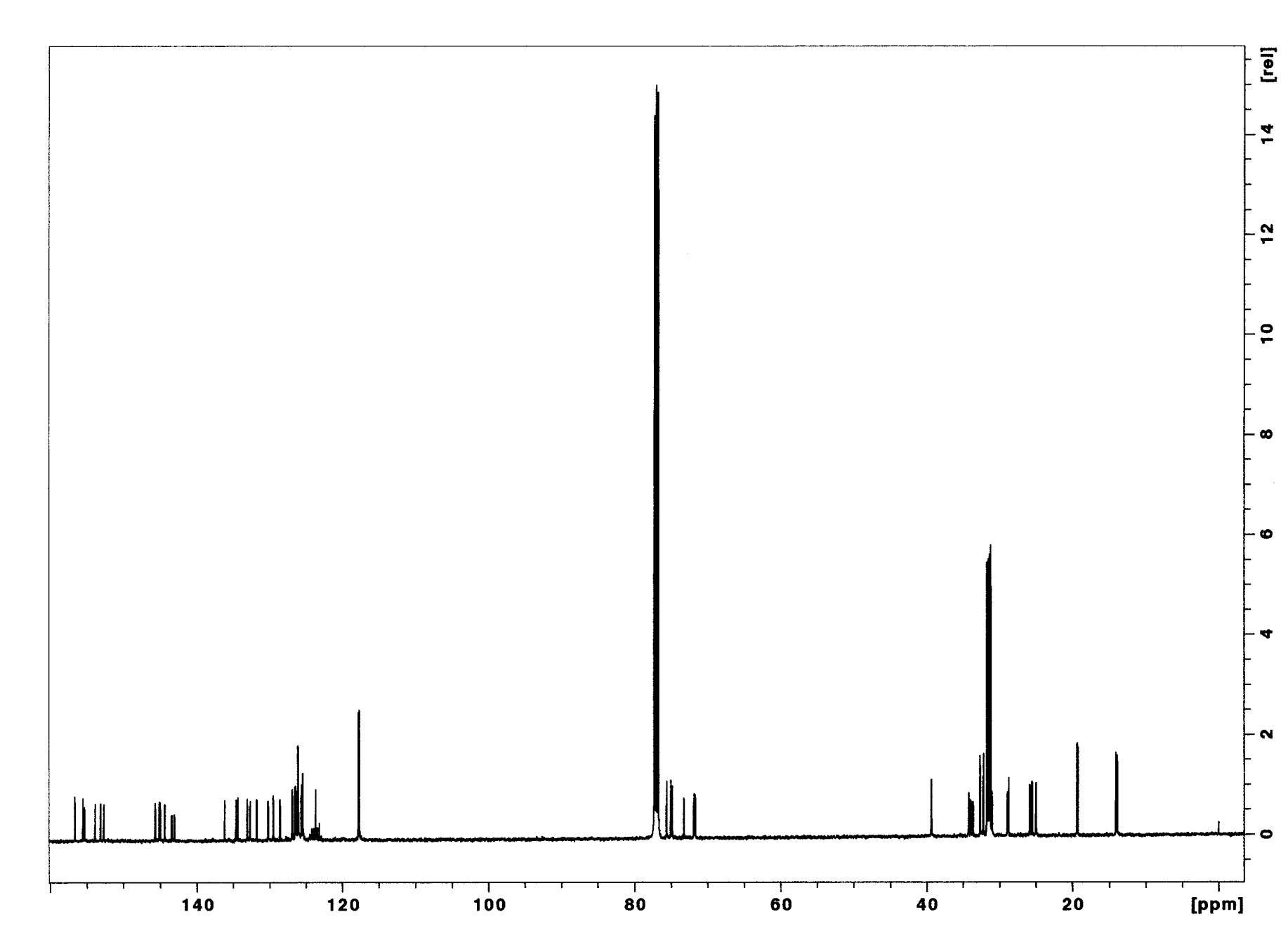


**Figure S6.** ^13^C NMR spectrum of CF_3_-Phurea **5b**.


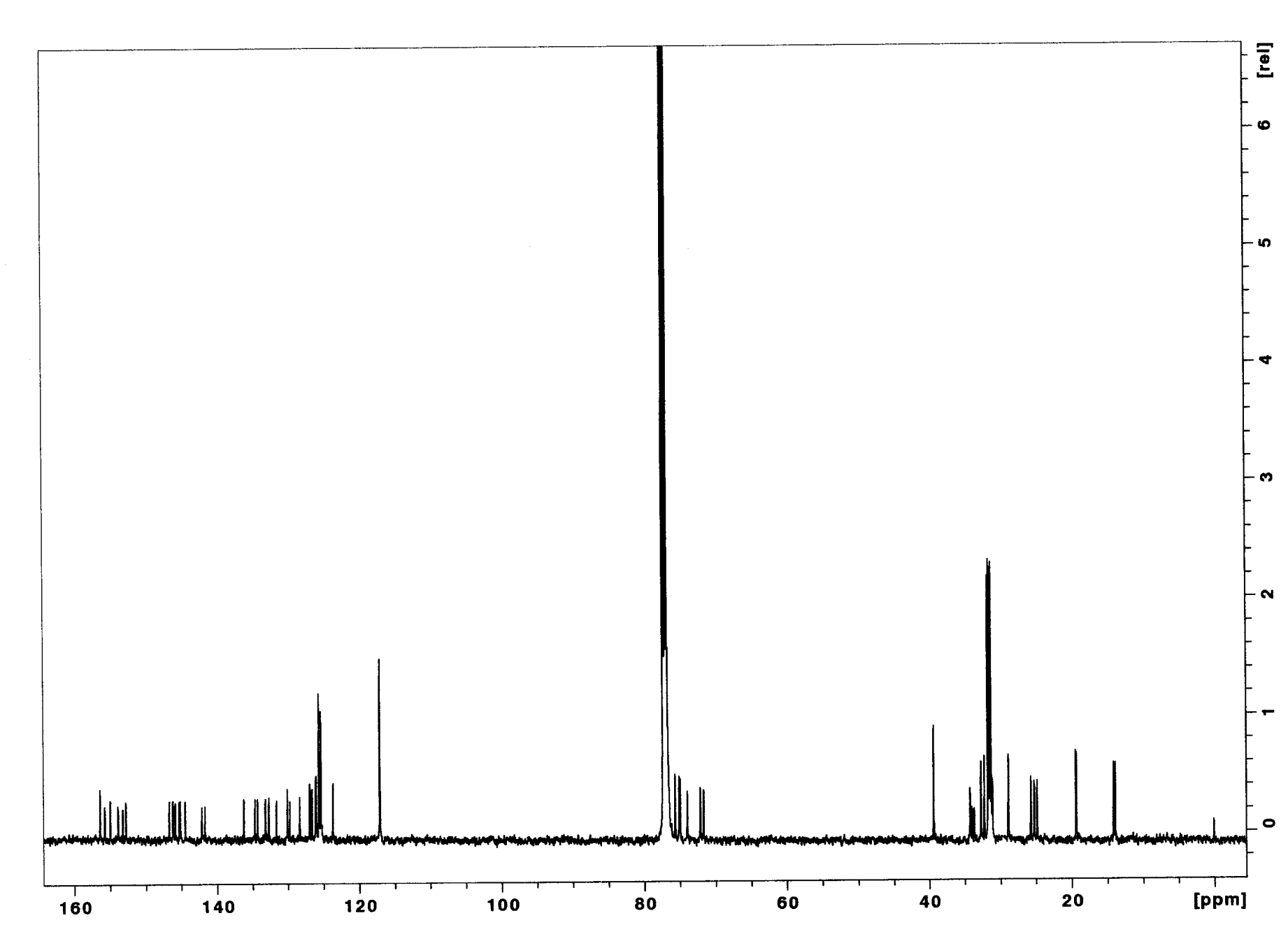


**Figure S7.** ^13^C NMR spectrum of NO_2_-Phurea **5c**.
